# Effect of Bovine Milk Peptides on Cell Inflammation, Proliferation and Differentiation: Milk Potential Benefits Are Preserved in an Unconventional Cow Feeding Strategy

**DOI:** 10.3390/biology12091162

**Published:** 2023-08-23

**Authors:** Costanza Cicchi, Paolo Paoli, Alessandra Modesti, Federica Mannelli, Federica Scicutella, Arianna Buccioni, Carolina Fontanarosa, Simone Luti, Luigia Pazzagli

**Affiliations:** 1 Department of Experimental and Clinical Biomedical Sciences, University of Florence, Viale Morgagni 50, 50134 Florence, Italy; c.cicchi@student.unisi.it (C.C.); paolo.paoli@unifi.it (P.P.); alessandra.modesti@unifi.it (A.M.); luigia.pazzagli@unifi.it (L.P.); 2 Department of Agricultural, Food, Environmental and Forestry Sciences and Technologies, University of Florence, Piazzale delle Cascine 18, 50144 Florence, Italy; federica.mannelli@unifi.it (F.M.); federica.scicutella@unifi.it (F.S.); arianna.buccioni@unifi.it (A.B.); 3 Department of Chemical Sciences, University of Naples Federico II, 80138 Naples, Italy; carolina.fontanarosa@unina.it; 4 Consorzio Interuniversitario I.N.B.B., Viale Medaglie D’Oro, 00136 Rome, Italy

**Keywords:** feeding strategies, olive oil pomace, bioactive peptides, antioxidant, anti-inflammatory, antiproliferative activity, cell differentiation induction

## Abstract

**Simple Summary:**

Transitioning to sustainable food systems includes the reuse of agricultural by-products as alternative feed ingredients in farms with the aim to increase animal efficiency while reducing the environmental footprint. Olive oil pomace is a waste product obtained during olive oil milling, and its chemical composition makes it a suitable source of fiber, fat, protein and polyphenol in the diet of lactating cows. The inclusion of olive oil pomace in the dairy cow diet did not impair nutrient degradability at the rumen level and animal performances and increased the amount of milk polyunsaturated fatty acids. In this study, the milk protein content from cows fed with a diet supplemented with olive oil pomace was considered; a simulation of gastrointestinal digestion was used to obtain peptides from milk proteins. The peptides were characterized for their antioxidant and anti-inflammatory activity and for their ability to induce differentiation and reduce proliferation in human colon cancer cells. No significant differences were detected between peptides derived from cows fed with a commercial diet; therefore, milk from cows fed with olive oil pomace can be safely used in humans, having nutritional features like (if not superior) traditional milk, but it can be obtained with less use of hay.

**Abstract:**

Animal feeding through the reuse of agro-industrial by-products in one of the ultimate goals of sustainable agriculture. Olive oil pomace (OOP) produced as a waste product during olive oil milling has been used as an ingredient in the diet for Holstein lactating cows. Recent findings have shown no decrease in animal performance, feed intake or detrimental effect on rumen microbiota. In contrast, an improvement in C18 polyunsaturated fatty acids has been observed. In this work, the milk protein content from cows fed a commercial diet (CON) or an experimental one supplemented with OOP was determined and compared, and the peptides derived from the simulated gastrointestinal digestion of raw milk were analyzed. After fractionation via RP-HPLC, peptides were characterized for their biological activity on different cell lines. The ability to reduce both the intracellular ROS content and the expression of inflammatory markers, such as Cyclooxygenase, isoenzyme 2 (COX-2) and inducible Nitric Oxide Synthase (iNOS), as well as the remarkable properties to induce cell differentiation and to slow down the proliferation of human intestinal cancer cells, enable us to define them as bioactive peptides. In spite of there being no observed significant difference between the healthy activity of CON and OOP peptides, the results allow us to broaden the knowledge about the biological activity of these bioactive peptides and to confirm that agro-industrial by-products may be successfully incorporated into the feeding strategy of dairy cows.

## 1. Introduction

Milk is a complete food that is important in the diet of many people worldwide, particularly in the Mediterranean, Western Europe and North America. In addition to being a rich source of carbohydrates, fatty acids, minerals and vitamins, milk is a good source of noble protein, with approximately 35 g/L. Milk proteins are divided into two broad categories: caseins, which contribute to about 80% of the total milk proteins and are organized into macromolecular micelles, and whey proteins, which are soluble, rich in essential amino acids and include alfa-lactalbumin, lactoferrin, immunoglobulins and enzymes [1]. In addition to their nutritional and functional value, milk proteins are an exceptional source of bioactive peptides that may have beneficial effects on human health. In fact, gastrointestinal digestion through proteolytic enzymes, microbial fermentation or food processing releases peptides of about 2–20 amino acids that become equipped with several biological activities [2]. Commercial proteases have been successfully tested for the production of bioactive hydrolysates from milk, mainly derived from caseins and, more recently, from whey proteins [3]. In recent years, many articles have been published dealing with the bioactive peptides produced from various food sources, like meat, fish, algae and plants, via protease activity or fermentation [4]. Milk-derived bioactive peptides exert multiple actions, including antimicrobial, anti-inflammatory, antioxidant, blood-pressure-lowering, anti-proliferative and anti-diabetic activity [2,5]. Interestingly, antioxidant and anti-inflammatory activities are often present in the same amino acid sequence as a witness to the interdependence between oxidative stress and inflammation, which act in synergy and may be the basis for other pathological conditions, such as aging-related diseases, atherosclerosis and cancer, that often share common pathological mechanisms, including abnormalities in inflammatory responses and oxidative stress [6,7].

Due to the wide range of putative healthy effects of milk, as a complete food and as a source of bioactive peptides, many studies are underway to improve both the quality of bovine milk and the dairy cow efficiency [8,9]. In this respect, animal genetics and nutrition may play an important role in the milk protein content and, consequently, in the bioactive peptides derived therefrom [10]. In the last few decades, the requirement for a more sustainable food production chain became mandatory, and the use of unconventional ingredients in the diet of animals may represent an opportunity to reduce the environmental impact and the feed vs. food competition, as well as to valorize the by-products from other production chains in congruency with the principles of the circular economy.

Hence, recent studies have investigated new alternative feeding strategies based on the reuse of agro-industrial by-products [11]. 

Most edible by-products are characterized by the presence of functional molecules able to modulate ruminant fermentation, improving the quality of milk or meat as well as reducing gas emissions. Among these, OOP from the olive oil milling process has recently been used as a promising nutritional supplement in the diet of Holstein lactating cows because of its richness in thyrosol, hydroxythyrosol and oleoeuropein, which improve milk quality due to the enrichment in polyunsaturated fatty acids [12]. Polyphenols are antimicrobials able to interfere with the activities of rumen microbial communities. Their ability in complexing feed proteins in the rumen is well known, limiting the degradation of the protein quite excessively with respect to the dietary energy fraction. The higher quantity of protein bypass optimizes the nitrogen retention in cows, who may use it for the synthesis of milk proteins. However, the class of polyphenol compounds is very large, and their chemical property is highly variable and strongly linked to their structure and solubility. Several authors found that the presence of several kinds of polyphenols in the diet of dairy ruminants may affect casein synthesis, increasing the Casein Index, the expression of the percentage of casein with respect to the total protein, indicating that a gene expression modulation or the availability of several amino acids occurred [13]. However, few studies on the effect of OOP on milk proteins are reported in the literature.

In a previous study analyzing the effect of OOP supplementation in a dairy cow feeding strategy, we demonstrated that it did not affect dry matter intake, rumen percentage of degradability and milk production. On the contrary, the milk’s nutritional quality was improved by increasing several important functional fatty acids, such as linoleic acid, conjugated linoleic acid, oleic acid and vaccenic acid [12].

Hence, in the present work, we used the same experimental model to investigate the effects of OOP supplementation on the protein content and the bioactive peptides obtained via the simulated gastrointestinal digestion of milk, aiming to deepen the impacts of a diet supplemented with OOP in respect to milk from cows fed with a commercial diet (CON).

## 2. Materials and Methods

### 2.1. Materials

Proteases, culture media and general reagents were purchased from Sigma-Aldrich, St. Louis, MO, USA, unless otherwise stated. All organic solvents used for HPLC analysis were high-performance liquid chromatography grade and were purchased from Sigma-Aldrich. Antibodies: anti Actin 13E5 (Cell Signaling Technology, Inc., Danvers, MA, USA), Anti-COX2 C-20 and β-tubulin (F-1) (Santa Crux Biotechnology, Dallas, TX, USA), and anti-iNOS PA1-036 (Thermo Fisher Scientific, Waltham, MA, USA). 

### 2.2. Recovery of Milk Samples

Milk was obtained from 40 lactating cows randomly assigned to two groups. Briefly, 20 cows were fed with CON, and 20 cows were fed with a diet supplemented with 8% OOP. The two diets were isoproteic and isoenergetic. The trial lasted four weeks after two weeks of adaptation to the experimental diets. Milk was individually and weekly sampled to monitor proximate profile. On the 29th day, 30 mL of milk from each cow was collected (20 samples from CON group and 20 samples from OOP group) and stored at −80 °C until analysis. More details on diet composition, analysis and animal performances are reported in Scicutella et al. [12]. Protein content was determined using the bicinchoninic acid method (BCA, Pierce Chemical, Rockford, IL, USA) and the Bradford assay (BioRad, Hercules, CA, USA). Protein profile of milk samples was determined by using 4–20% sodium dodecyl sulphate polyacrylamide gel electrophoresis (SDS-PAGE, mini-protean precast TGX gels, BioRad, Hercules, CA, USA).

### 2.3. Production of Peptides via Enzymatic Digestion

Cow milk digestion was performed according to Tagliazucchi et al. [14], with minor modifications. Thus, 30 mL of milk both from CON and OOP cows (1.5 mL from each animal) was defrosted and assayed for protein concentration. Protein assay was performed (both on the milks from each cow and on the 30 mL of collected bulk milks) using both the BCA and the Bradford method according to the literature [15]. Peptides were obtained via the use of a mixture of enzymes to simulate gastrointestinal digestion following the protocol of Minekus et al. [16], with some modifications. Firstly, pH was lowered to 2.5 through addition of 1M HCl (900 μL); then, pepsin was added to a final concentration of 2000 U/mL, and the mixtures were incubated at 37 °C for 2 h under stirring. After pepsin digestion occurred, firstly, the pH was increased to 8.2 with 20% (*w*/*v*) Na_2_CO_3_, then 0.8 mg/mL of pancreatin was added and the mixtures were kept in incubation under continuous stirring for 2 h at 37 °C. To separate peptides from the undigested proteins, five volumes of cold acetone were added, the samples were kept overnight at −80 °C and, finally, they were centrifuged at 3500 rpm (1300× *g*) at 4 °C for 15 min. The supernatants were recovered, freeze-dried and re-dissolved in 2 mL of phosphate-buffered saline pH 7.4. Lipids and other insoluble molecules were removed through fractionate precipitation using a solution of chloroform/methanol (2/1, *v*/*v*) that was added to peptides in 1:2.5 ratio according to the Folch method [17]. The mixture was kept overnight at −20 °C and then centrifuged at 2700× *g* for 5 min at 4 °C. The supernatant containing the peptides was recovered and further freeze-dried. Peptides, both from CON milk and peptides from OOP milk, were resuspended in 0.05% TFA (trifluoroacetic acid) in water and further assayed for protein concentration and HPLC analysis.

### 2.4. HPLC Fractionation to Collect Peptides

Aliquots of about 200 µg of each sample were centrifuged at 10,000× *g* for 10 min and then applied to HPLC (Ultimate 3000, Thermo Scientific, Waltham, MA, USA) equipped with a C18 reverse-phase column (Kinetex, 4.6 × 250 mm, 5 μm, 100 Å, Phenomenex, CA, USA) and a UV detector operating at 214 nm. Elution was carried out at 0.8 mL/min flow rate using a water/acetonitrile (CH_3_CN) gradient containing 10 mM TFA. Solvent A: H_2_O + 10 mM TFA; Solvent B: CH_3_CN + 10 mM TFA. Gradient: 0–10 min 0%B, 10–25 min 0–15%B, 25–60 min 15–50%B, 60–65 min 50–100%B, 65–73 min 100%B, 73–75 min 100–0%B. Eluted peptides were manually fractionated from 15 to 50 min retention time. Solvents were removed from collected samples via freeze-drying, and peptides were re-dissolved in sterile PBS and subjected to further characterization. 

### 2.5. Cell Cultures for Ex Vivo Assays

RAW 264.7 murine macrophage and Human colorectal adenocarcinoma Caco-2 were purchased from Sigma-Aldrich. RKO colon carcinoma cell line was purchased from ATCC. Cells were cultured in standard conditions under humidified atmosphere (5% CO_2_, 37 °C) using Dulbecco’s Modified Eagle Medium (DMEM) (Raw 264.7, RKO) and Minimum Essential Medium (MEM) (Caco-2) supplemented with 10% (*v*/*v*) fetal bovine serum (FBS), 1 mM glutamine and 100 μg/mL penicillin/streptomycin. Cells were routinely passed twice a week (RAW 264.7 and RKO) and once a week (Caco-2).

RAW cells are used to detect antioxidant and anti-inflammatory activities of peptides according to the literature [18,19]; anti-proliferating activity and induction of differentiation are assayed on RKO and Caco-2 cells, respectively [20]. 

### 2.6. Cell Viability and Cell Viability Recovery Assay

Cell viability was assessed by treating RAW 264.7 and Caco-2 cultured cells with milk-derived peptides. RAW 264.7 cells were plated in a 24-well plate in 0.5 mL fresh medium (DMEM) at a density of 2.5 × 10^5^ cells per well, and Caco-2 cells were plated in 0.5 mL fresh medium (MEM) at a density of 5x10^4^ cells per well. After 24 h, peptides were added to cultured cells at a final concentration of 0.2 μg/μL, 0.1 μg/μL and 0.05 μg/μL. The cells treated with PBS were used as control. After 24 h, a crystal violet assay was performed, and absorbance was measured at 595 nm according to Feoktistova et al. [21]. Then, 200 μL of crystal violet solution was added to each well and incubated at room temperature with shaking for 30 min. Crystal violet was discarded, and each well was washed three times with distilled water. Cells were lysed in 2% SDS, and absorbance was measured at 595 nm. Cell viability was expressed as % absorbance compared to PBS-treated cells. 

RAW 264.7 cells were also used to detect the recovery of cell viability upon an inflammatory stimulus. Cells were plated on 24-well plate at a density of 5 × 10^5^ cells per well. After 24 h, peptides were added at a final concentration of 0.05 μg/μL, and they were incubated for 1 h at 37 °C. After incubation, lipopolysaccharide (LPS) 1 μg/mL was added to the medium, and cells were incubated for 24 h [22]. Cells without the addition of peptides were used as the negative control; cells treated with 1 µg/mL LPS were used as the positive control. Cell viability recovery was expressed as % absorbance compared to the positive control.

### 2.7. Reactive Oxygen Species (ROS) Assay

Experiments were performed in 24-well plates, using RAW 264.7 cells at a density of 5 × 10^5^ per well. Cells were treated with milk peptides at a final concentration of 0.05 μg/μL. After 1 h, cells were co-incubated with 1 μg/mL LPS to induce ROS production [23]. Cells stressed with LPS were used as positive control, while stressed cells treated with 0.05 mM ascorbic acid were used as negative control. Intracellular ROS production was measured through oxidation of the fluorescent probe H2DCF-DA (2′,7′-dichlorodihydrofluorescein diacetate) 10 μM in DMSO according to Luti et al. [24]. The probe was added to the medium, and cells were incubated for 1 h. After incubation, ROS production was detected using a Biotek Synergy H1 Plater Reader (Agilent, Santa Clara, CA, USA) by measuring fluorescence at λ_ex_ 485 nm and λ_em_ 538 nm. Fluorescence signal was normalized via protein content determined using the BCA assay. The values were determined as fluorescence intensity units and the results expressed as a percentage of the reduction in ROS formation.

### 2.8. Immunoblot Detection of COX2 and iNOS

RAW 264.7 cells were seeded and treated as described in 2.6. Further, 2 mM acetylsalicylic acid was used as negative control. Cells were lysed in RIPA (Radioimmuno precipitation assay) buffer, and protein content was determined via the BCA assay. Then, 20 μg proteins were separated by 10% SDS-PAGE and transferred to PVDF membranes. The membranes were blocked with 5% dry milk in PBS and 0.1% Tween for 1 h at room temperature. Membranes were incubated overnight with primary antibodies at 1:1000 at 4 °C.

Membranes were washed twice and incubated with horseradish peroxidase-conjugate secondary anti-mouse antibodies (SantaCruz Biotechnology, Dallas, TX, USA) diluted 1:5000 and secondary anti-rabbit antibodies (Invitrogen, Waltham, MA, USA) diluted 1:2500 in milk (PBS, 0.1% Tween) for 1 h at room temperature. After washing, chemiluminescence was detected using an ECL kit (GE HealthCare, Chicago, IL, USA) using an Amersham Imager 680 (GE HealthCare Life Sciences, Chicago, IL, USA), and results were expressed as percentage of COX2 and iNOS expression compared to LPS-stimulated cells. 

All values were normalized on actin or tubulin signal. 

### 2.9. Cell Proliferation Assay

Effect of milk-derived bioactive peptides on cell proliferation rate was determined on colon carcinoma RKO cell line [25,26]. RKOs were plated in a 24-well plate at a cell density of 20,000 cells per well. After 24 h, cells were treated with 0.05 and 0.1 μg/μL peptides for 24 and 48 h. Cells were then detached using Trypsin-EDTA and 10 μL cell suspension was transferred to a Burker chamber. Cells were counted using a light optical microscope, and results were expressed as percentage of cell proliferation compared to PBS-treated cells. To highlight the synergistic effect of milk-derived peptides and 5-fluorouracil (5-FU), RKO cells were plated in a 24-well plate at a cell density of 20,000 cells per well. After 24 h, cells were treated with a combination of 0.1 μg/μL peptides and 1, 2.5 and 5.0 μM 5-FU. After 48 h, cells were detached and counted as described below. 

### 2.10. Cell Differentiation 

Impact of bioactive peptides on cell differentiation was determined on Caco-2 cells according to Klepinina et al. [20]. Cells were seeded on 24-well plates, grown until confluency and treated with 0.1 μg/μL milk-derived peptides and 5 mM sodium butyrate. Cells treated only with 5 mM sodium butyrate were considered as positive control. After 72 h, cell monolayers were washed with PBS and 0.5 mL of 2.5 mg/mL pNPP (p-nitro-phenyl-phosphate) solution in 100 mM Glycine; 2 mM MgCl_2_ (pH 9.8) was added to each well. Cells were then incubated at 37 °C for 20 min, 100 μL aliquots were transferred to a 96-well plate and 100 μL NaOH 0.5 M was added to completely stop the reaction. Absorbance was read at 405 nm using a Biotek Synergy H1 plate reader (Agilent, Santa Clara, CA, USA). Alkaline phosphatase activity was expressed as nmol p-NPP formed in 0.5 mL reaction solution, and enzymatic activity was normalized on protein content measured by the Bradford assay. Results are expressed as percentage of alkaline phosphatase activity compared to PBS-treated cells. 

### 2.11. Mass Spectrometry Analysis 

Peptide samples were loaded via an autosampler (Surveyor MS Pump Plus and Micro AS) onto a Michrom C18 Captrap and then were introduced directly into a Orbitrap LTQ-Velos MS (Thermo Fisher Scientific, Surrey, UK) via a fused silica C18 capillary column (75 µm, 43 mm; Nikkyo Technos CO, Tokyo, Japan) and a nano-electrospray ion source. The mobile phase comprised H_2_O with 0.1% formic acid (buffer A) and 100% acetonitrile with 0.1% formic acid (buffer B). The gradient ranged from 5% to 30% buffer B in 95 min, followed by 30% to 60% B in 15 min and a step gradient to 85% B for 5 min with a flow of 0.42 μL min^−1^. Finally, the system returned to the initial conditions of 5% B. FTMS full-scan mass spectra (from 450 to 1600 m/z) were acquired with a resolution of r = 60,000. This was followed by data-dependent MS/MS fragmentation in centroid mode, the most intense ion from the survey scan using collision-induced dissociation (CID) in the linear ion trap: normalized collision energy 35%; activation Q 0.25; electrospray voltage 1.5 kV; capillary temperature 200 °C; and isolation width 2.00. This MS/MS scan event was repeated for the top 20 peaks in the MS survey scan; the targeted ions were then dynamically excluded for 30 s. Singly charged ions were excluded from the MS/MS analysis; Xcalibur software version 2.1.0 SP1 build 1160 (Thermo Fisher Scientific, Surrey, UK) was used for data acquisition. The acquired MS/MS spectra were converted in m/z Data (.XML) format and used for protein identification with a licensed version of MASCOT software (www.matrixscience.com; accessed on 13 December 2021) version 2.4. with 10 ppm MS tolerance and 0.6 Da MS/MS tolerance.

### 2.12. Statistical Analysis

Data are presented as means ± standard deviation (SD) from at least three experiments. Normal distribution of data was analyzed using the Shapiro–Wilk test. 

Statistical analysis was performed using t-Student or one-way ANOVA for normally distributed data sets, while the non-parametric Wilcoxon signed-rank test was used for non-normal distributions (iNOS data set). Tests were performed in GraphPad Prism 5, setting statistical significance at *p* < 0.05.

## 3. Results

### 3.1. Determination of Protein Content and Purification of Bioactive Peptides

**Figure 1:**
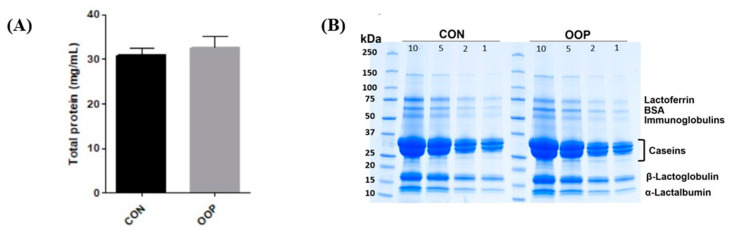
Protein content in the pooled fractions of milk. (**A**) Bradford assay on milk from cows fed with commercial diet (CON, dark grey) and fed with olive oil pomace (OOP, light grey). (**B**) 4–20% SDS-PAGE of CON (left panel) and OOP, (right panel). Numbers on each line are referred to the µg of applied protein. Gels were stained with Comassie Brilliant Blue. (*n* = 8).

The protein contents of CON (30.74 ± 1.7 mg/mL) and OPP milk (32.60 ± 2.5 mg/mL), respectively, were not significantly different (*p* = 0.592), and they fit with the protein amount of commercial non-skimmed milk (Figure 1). Moreover, the SDS-PAGE analysis revealed no qualitative differences in the protein content of CON- and OOP-derived samples, suggesting that the addition of a waste product to the diet of lactating cows does not affect the protein content in the milk.

**Figure 2:**
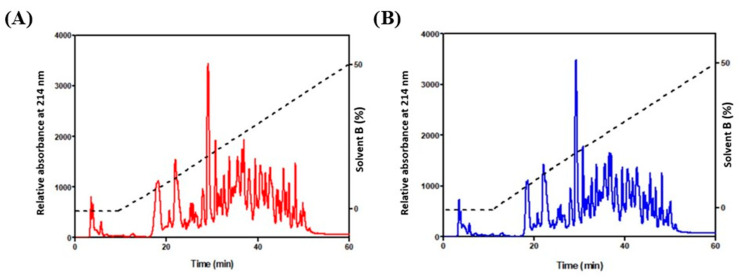
HPLC fractionation of peptides. (**A**) CON milk (red line) and (**B**) OOP milk (blue line). Column: Kinetex, 4.6 × 250 mm, 5 μm, 100 Å; flow rate 0.8 mL/min. Gradient (dotted line): water/CH_3_CN in10 mM TFA.

Through the simulation of gastrointestinal digestion, we obtained milk-protein-derived peptides, and an SDS-PAGE analysis was performed on digested samples, confirming protein cutting. To further purify the obtained peptides, aliquots of these samples (about 200 µg for each) were injected into a reverse-phase column, and eluted peaks were collected in two fractions, identified as “control” (CON) and “olive oil pomace peptides” (OOP) peptides (Figure 2). They were freeze-dried, resuspended in PBS at 1 mg/mL and used to evaluate their biological activity in cultured cells. 

### 3.2. Antioxidant and Anti-Inflammatory Effects of CON and OOP Peptides on RAW Cells

#### 3.2.1. Cell Viability

**Figure 3:**
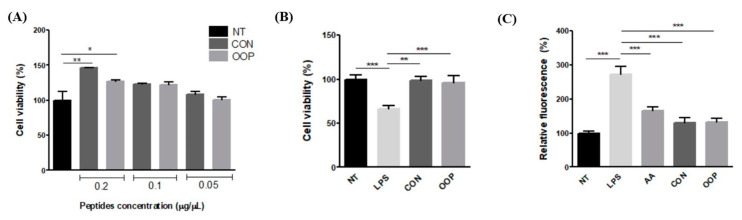
Effect of CON and OPP peptides on cell viability and ROS production. (**A**) Effect of peptides on cell viability. RAW 264.7 cells were incubated with the peptides (0.2–0.05 mg/mL) for 24 h. Cell viability was evaluated using crystal violet test. (**B**) Recovery of cell viability: cells were pre-incubated with 0.05 μg/μL peptides for 1 h and then with LPS for 24 h. (**C**) Measurement of ROS production: Cells were treated with 0.05 μg/μL of CON and OPP peptides or with 0.05 mM ascorbic acid. After 1 h, cells were co-incubated with 1 μg/mL LPS. NT: Negative Control, untreated cells (black bar); LPS: positive control, cells treated with LPS alone; AA ascorbic acid, negative control: cells treated with ascorbic acid and LPS. CON peptides: dark gray; OOP peptides: light gray. Results are the mean (±SD) of four different experiments performed in duplicate. Statistical analysis was performed for each sample vs. the NT value (**A**) and vs. the LPS value (**B**,**C**) (* *p* < 0.05; ** *p* < 0.01, *** *p* < 0.001).

The results show that none of the used concentrations impair cell viability, thereby confirming that CON and OOP peptides have no toxic effect; on the contrary, a positive effect on viability can be observed in cells treated with 0.1 and 0.2 µg/µL. The cellular viability of cells treated with 0.05 µg/µL of peptides is the same as that of untreated cells, and it was used for the recovery of cell viability determination (Figure 3A). To this end, cells were pre-treated with peptides and then treated with LPS to reduce the cell viability as a consequence of the activation of inflammatory pathways and of the synthesis of reactive oxygen species. Figure 3B clearly shows that both CON and OOP samples have the ability to protect against LPS-induced damage in a significant manner, maintaining cell viability like that of untreated cells. 

#### 3.2.2. Antioxidant Activity 

To evaluate if the recovery of cell viability can be partially due to the reduction in intracellular ROS concentration, the fluorescent probe DCFH-DA was used on stressed cells treated with peptides. The reduction in free radicals induced by CON and OOP peptides was highly significant when compared to the LPS value, and it was quite similar to that detected in cells treated with the antioxidant molecule ascorbic acid (*p* > 0.05 ascorbic acid vs. peptides, Figure 3C). The results suggest that peptides are able to scavenge the ROS produced upon LPS stress, or, alternatively, they can reduce the synthesis by lowering the inflammatory state induced by the treatment.

#### 3.2.3. Anti-Inflammatory Activity

**Figure 4:**
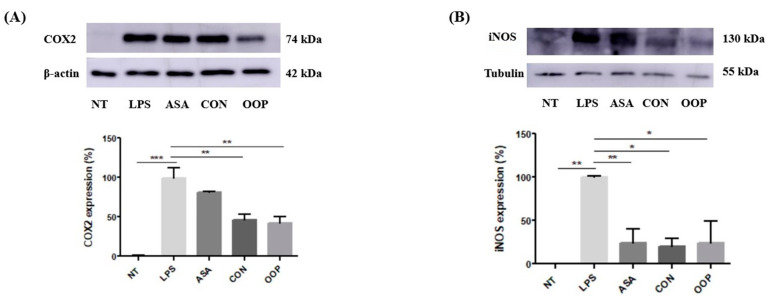
Anti-inflammatory activity of CON and OPP peptides. RAW 264.7 cells were pre-incubated with 0.05 μg/μL peptides for 1 h and then with 1 µg/mL LPS for 24 h. (**A**) COX2 expression analysis: upper panel: Western blot analysis of a representative experiment; lower panel: relative protein level from Western blot data. Actin was used as a loading control. (**B**) iNOS expression analysis: upper panel: Western blot analysis of a representative experiment; lower panel: relative protein level from Western blot data. Tubulin was used as a loading control. NT: Negative Control, untreated cells; LPS: positive control, cells treated with LPS alone; ASA negative control: cells treated with acetylsalicylic acid and LPS. Results are the mean (±SD) of two different experiments performed in triplicate. Statistical analysis was performed for each sample vs. the LPS value (* *p* < 0.05; ** *p* < 0.01, *** *p* < 0.001).

The anti-inflammatory activity of CON and OOP peptides was checked by detecting the expression levels of iNOS (inducible Nitric Oxide Synthase) and COX-2 (cyclooxygenase, isoenzyme 2), which were chosen as downstream markers of the anti-inflammatory response. RAW cells were pre-treated with CON and OOP peptides for an hour and then incubated with LPS for a further 24 h before the analysis; specific monoclonal antibodies were used to detect proteins in cell lysates via Western blot (Figure 4). The results indicate that, as expected, COX2 remains unexpressed under normal conditions and rises in inflammation, while the pretreatment with peptides is able to strongly reduce COX2 expression to values lower than those obtained with acetylsalicylic acid that was used as a negative control (Figure 4A). The same results were obtained by detecting iNOS expression (Figure 4B) to corroborate the hypothesis that bioactive peptides may have a beneficial effect on cultured cells, by reducing either oxidative response or inflammation, processes that are closely interdependent.

### 3.3. Antiproliferative and Differentiation Induction Activities of CON and OPP Peptides

In addition to the antioxidant and anti-inflammatory properties, other potential biological activities of the peptides were analyzed in intestinal cells, with which they can physiologically come into contact. 

#### 3.3.1. Antiproliferative Effect on RKO Cells

**Figure 5:**
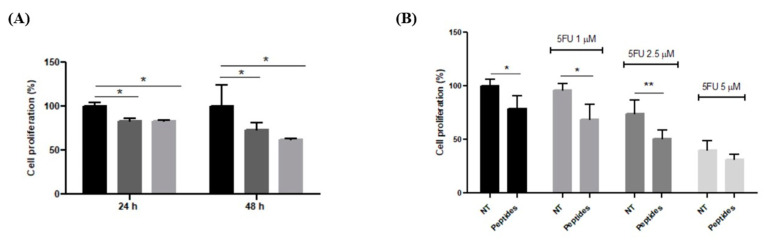
Effect of peptides on RKO cell proliferation. (**A**) Cell proliferation in presence of 0.1 µg/µL of CON (dark-grey bar) and OOP (light-grey bar) peptides. Results are expressed as percentage of cells compared to the non-treated cells (black bar). (**B**) Cell proliferation after 48 h of incubation with 0.1 µg/µL of peptides in presence of different concentrations of 5-FU. Results are the mean (±SD) of two different experiments performed in triplicate. Statistical analysis was performed for each sample vs. the NT value (* *p* < 0.05; ** *p* < 0.01).

The antiproliferative activity of CON and OPP peptides was tested on RKO cells (Human colorectal cancer cells) that are currently used to determine the anticancer activity of several compounds. The inhibitory effects of the peptides against the proliferation of RKO cells were concentration- and time-dependent: 0.05 µg/µL of peptides after 48 h of incubation determined a 10% reduction in proliferation that was not statistically significant. However, at 0.1 µg/µL, a difference in the proliferation becomes noticeable, and the effect is more evident after 48 h than 24 h (Figure 5). The antiproliferative effect of the peptides was also determined in the presence of 5-FU, a well-known cytotoxic chemotherapeutic agent often used in combination with other antineoplastic drugs, lowering its toxicity. Further, 1.0–2.5–5.0 µM 5-FU was used in co-treatment with 0.1 µg/µL of peptides for 24 and 48 h. At the lowest concentration of 5-FU used, cell proliferation was reduced by about 30% when peptides were used in cotreatment with 5-FU; on the contrary, fluorouracil alone was unable to affect the number of cells present in the well after 48 h of incubation. The synergistic effect was more evident with 2.5 µM 5-FU (*p* < 0.01); instead, the 5.0 µM concentration causes a deep cytotoxic effect, hiding the synergic effect of the peptides. 

#### 3.3.2. Differentiation of Caco-2 Cells

**Figure 6:**
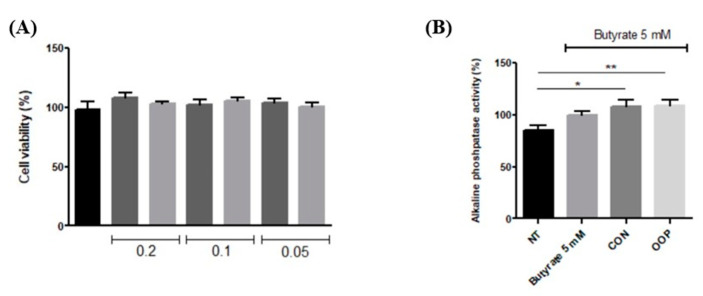
Caco-2 cells differentiation with CON and OOP peptides. (**A**) Cell viability of differentiated Caco- 2 cells treated with different peptide concentrations (0.2–0.05µg/µL). Viability was determined by Crystal Violet. (**B**) Differentiation of Caco-2 cells. Monolayers of Caco-2 cells were treated with 5 mM butyrate. Differentiation is determined as the ratio between alkaline phosphatase activity and total protein content. Results are reported as a percentage of the butyrate value. NT: non-treated cells (negative control). Results are the mean (±SD) of three different experiments performed in triplicate. Statistical analysis was performed for each sample vs. the NT value (* *p* <0.05; ** *p* < 0.01).

Caco-2 cells are currently used as a model system for the small intestinal epithelium; they are derived from human epithelial colorectal adenocarcinoma cells and can differentiate into monolayer cells after confluence. Different concentrations of samples were first tested to evaluate a possible effect on cell viability. The results show that none of the concentrations had an impact on cell viability. Thus, 0.1 μg/μL peptides was chosen as the condition to investigate cell differentiation. Sodium butyrate was used to induce cell differentiation, both alone and in the presence of CON and OPP peptides. As shown in Figure 6 butyrate is able to induce differentiation if compared to the non-treated cells, and pre-incubation with peptides induces a further increase in cell differentiation that was monitored by the alkaline phosphatase activity. Butyrate induces differentiation, but significant values are reached only with the pre-incubation with peptides to suggest a possible synergistic effect.

### 3.4. Identification of Bioactive Peptides from CON and OOP via Mass Spectrometry

**Figure 7:**
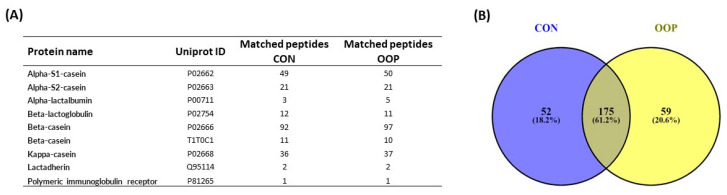
Mass spectrometry characterization of milk-derived peptides: (**A**) list of parent milk proteins releasing peptides; (**B**) Venn diagram of peptides in CON (left panel) and in OOP (right panel) samples (https://bioinfogp.cnb.csic.es/tools/venny/ (accessed on 5 July 2023)). Numbers represent the amount of peptides in each sample identified via LC-MS analysis.

To further characterize CON and OOP content, we performed a mass spectrometry identification of peptides present in the samples using LC-MS. We identified a total of 286 peptides that are reported in Supplementary Table S1. They derived from a total of nine milk proteins: Alpha-S1 and S2 casein, Alpha and Beta-lactoglobulin, Beta-casein (2 isoforms), Kappa-casein, Lactadherin and Polymeric immunoglobulin receptor and the 87% of identified peptides derived from casein (Figure 7A). A Venn diagram was used to summarize the similarities and differences among CON and OOP in terms of peptide sequences (Figure 7B). The results clearly showed that 175 (61.2%) of the identified peptides share the same sequences (see Table S1 for detail), suggesting that the majority of peptides are derived from the same proteolytic cleavage. The results from the Venn diagram also allow for the identification of peptides that are unique for each sample (Table S1), and they represent 18.6% and 20.6% of the total peptides for CON and OOP, respectively.

## 4. Discussion

Animal feeding represents an efficient tool for the sustainability of food production, and part of the efficiency in converting feed into end products depends on the quality of the diet. Consistent with the 3Rs (Reuse, Recycle and Reduce) principle, a feeding strategy based on unconventional ingredients, such as those derived from agricultural bio-waste, could be an interesting opportunity. Most edible by-products contain bioactive molecules that may interfere with rumen microbial activities or with the host animal metabolism. The feeding of ruminants with diets enriched in polyphenols is increasingly attracting the attention of nutritionists. The scientific literature reports that polyphenols can show either positive or negative properties when fed to a domestic animal. Polyphenols are able to complex proteins and free amino acids, either at the rumen or gut level, can exert antimicrobial activities, can modulate the expression of several genes [27] and interfere with the syntheses of milk protein [28].

In a previous study analyzing the effect of OOP supplementation in a dairy cow feeding strategy, we demonstrated that it did not affect dry matter intake, rumen percentage of degradability and milk production. On the contrary, the milk’s nutritional quality was superior by increasing several important functional fatty acids, such as linoleic acid, conjugated linoleic acid, oleic acid and vaccenic acid [12].

In this context, the purpose of this work was to characterize other important components of milk, such as protein and its derived peptides produced during digestion in the human gastrointestinal system.

In spite of the ISO standard method for protein content determination being the Kjeldahl method, the Bradford assay is, in fact, a quick and accurate methodology [15], and it has been used for both milk and peptide fractionation, giving results that are in the range of commercial whole milk. The protein content in milk from OOP cows is higher than that recovered in CON milk, but the values are not significative, probably because of the low number of assays performed (*n* = 8). 

Actually, it has been reported that milk proteins are a source of peptides that show beneficial health effects, including immunomodulation, antihypertension, antithrombotic activity and antimicrobial activity [5]. Peptides can be usually produced through fermentation or enzymatic hydrolysis [29]; here, we chose to add pepsin and pancreatin to the raw material, without any fractionation or precipitation of milk proteins, in order to simulate gastrointestinal digestion, which occurs naturally following the ingestion of dairy products. Subsequently, peptides produced from both CON and OOP milk were fractionated via methanol/chloroform extraction and purified on RP-HPLC. Although the benefits of bioactive peptides from milk proteins are known [1], this work allowed us to analyze a large panel of activity of a peptide mixture produced through the simulation of natural gastrointestinal digestion, using concentrations that can approximately simulate the amounts of peptides that arrive to the intestinal cells following the ingestion of a portion of milk according to Caira et al. [30]. RAW 264.7 murine macrophages [24], human epithelial colorectal adenocarcinoma Caco-2 cells [31] and human colorectal cancer RKO cells [26,32] were used as model cells to reveal the health benefits of CON and OOP peptides. Firstly, cellular viability was determined using different amounts of peptides in the concentration range commonly used for antioxidant and anti-inflammatory activity determination [7]. Concentrations of 0.05–0.1 µg/µL are able to trigger an increase in cell vitality (Figure 3), and they were chosen to unravel the biological activities of peptides. Antioxidant and anti-inflammatory activity was detected on stressed RAW cells as the ability to reduce the synthesis of intracellular ROS and to reverse the expression of inflammatory markers, such as COX-2 and iNOS, confirming observations previously reported on peptides isolated from caseins and whey proteins or derived from fermented milk [33,34]. However, although no significant difference was observed between results obtained from CON and OOP peptides, it is of peculiar relevance that they not only reduce ROS like 0.05 mM ascorbic acid does but they also lower COX-2 and iNOS expression to levels detected in acetyl salicylic-acid-treated cells. 

As chronic activation of some inducible enzymes, such as iNOS and COX-2, plays pivotal roles in the development of certain inflammatory diseases that may initiate the oncogenesis process, and the use of anti-inflammatory drugs, such as aspirin, is proposed to prevent inflammation-associated carcinogenesis [35,36], both CON and OOP peptides were used to treat two lines of human intestinal cancer cells to highlight a putative positive effect on cell proliferation and differentiation. The peptides were used at concentration and incubation times usually reported in the literature [32,37], and they are capable of significantly reducing the proliferation of RKO cells. Moreover, a synergic effect on proliferation has been observed in the presence of 5-FU, a chemotherapy drug used, to date, for patients with colorectal cancer [38]. The synergism that would allow one to reduce the toxic dose of 5-FU is a recent promising strategy used to counteract colon cancer, and it was recently proposed to lower the toxic dose of 5-FU. To the best of our knowledge, the inhibition of proliferation and the synergic effect of milk peptides have never been observed before in a mixture of peptides, and they would open new scenarios in the research on the protective effect of dairy products in cancer treatment.

In addition to the role in cell proliferation, CON and OOP peptides have also been shown to have a positive effect on cell differentiation, thus counteracting the undifferentiating potential of cancer cells. The effect has been detected in Caco-2 cells in the presence of butyrate, which is proven to have beneficial effects on colon inflammation and cancer [39]. OOP peptides appear to have a greater effect on cell differentiation when compared to values obtained from CON peptides, even if the differences are not statistically significant.

We used mass spectrometry to unravel the composition of CON and OOP peptides, showing that they have quite a similar composition, but 20% of the identified peptides are unique for each sample. In particular, the analysis of the most intense signals obtained via mass spectrometry put in evidence some sequences unique for OOP peptides that have been previously characterized. For example, FVAPFPEVFG and SDIPNPI from α-1 casein and DVENLHLPLPL, PVVVPPFLQ PVLGPVRGPFPI and YPVEPF from β-casein have been characterized for their antioxidant, ACE inhibitor and opioid activities, respectively [40,41,42].

The overall results clearly show that the inclusion of OOP in the diet of lactating cows did not affect the protein milk content or quality and that peptides obtained from the hydrolysis of raw milk have the same healthy biological activity, whether they originate from the milk of cows fed a commercial diet or from the milk of cows fed olive oil pomace. Some peptides with relevant biological activity are recovered only in the OOP milk. Hence, further investigations are necessary to better understand the role of OOP in protein metabolism. 

## 5. Conclusions

Milk is one of the most nutritious foods: it is a rich source of carbohydrates, fatty acids and vitamins and contains high-quality proteins enriched in essential amino acids. Obtaining milk with better nutritional characteristics and, at the same time, being able to reduce the environmental footprint are the ultimate challenges of many research lines. In addition to the antioxidant and anti-inflammatory activity of the tested peptides, the present paper allowed us to highlight the other biological activities of milk-derived peptides not yet characterized in the literature, such as the effect on cellular proliferation and differentiation, and the topic deserves further study, considering that the effects have been observed precisely on intestinal cells, with which the peptides come into contact. However, despite the experiments showing that OOP dietary supplementation does not drastically affect either the composition or the biological activity of the peptides, on the other hand, it has been possible to demonstrate that by-products from olive oil processing may be successfully included in the feeding strategy of dairy cows. 

## Data Availability

The data presented in this study are available on request from the authors.

## Supplementary Materials

The following supporting information can be downloaded at: https://www.mdpi.com/article/10.3390/biology12091162/s1, Table S1: List of all identified peptides by LC-MS technique.
