# Supplementary material for: Effect of Bovine Milk Peptides on Cell Inflammation, Proliferation and Differentiation: Milk Potential Benefits Are Preserved in an Unconventional Cow Feeding Strategy
Source: Biology (Basel). 2023 Aug 23;12(9):1162. doi: 10.3390/biology12091162 (PMC10525111; doi:10.3390/biology12091162)
Supplement: Supplementary file 1 [file biology-12-01162-s001.zip › biology-2519632-supplementary.pdf]

Table S1: List of all identified peptides using LC-MS technique.

Protein ID° and name	Peptide sequence	Peptide mass	CON intensity	OOP intensity
**Peptides in CON and OOP**				
sp|P02666|CASB_BOVIN	GPFPIIV	741,44251	766200	1833400
P02662|CASA1_BOVIN	PVVVPPFLQPEVMG	1507,8109	1327700	1610000
sp|P02666|CASB_BOVIN	SLPQNIPPLTQT	1307,7085	1552600	1577900
sp|P02666|CASB_BOVIN	SLPQNIPPLTQTPVVVPPFLQPEV	2609,4469	160760	1577900
P02662|CASA1_BOVIN	PVVVPPFLQPE	1220,6805	1421700	1510300
sp|P02668var|CASK_VARI_BOVIN	PVVVPPFLQPEV	1319,7489	76675	1472400
sp|P02668var|CASK_VARI_BOVIN	YQGPIVLNPWDQV	1527,7722	1191200	1412700
sp|P02663|CASA2_BOVIN	LVYPFPGPIPN	1212,6543	925050	1200300
sp|P02668var|CASK_VARI_BOVIN	VYPFPGPIPN	1099,5702	1381100	1144300
P02662|CASA1_BOVIN	HLPLPL	688,4272	1035600	1064200
sp|P02666|CASB_BOVIN	PVVVPPFLQPEVM	1450,7894	2252200	1060300
sp|P02666|CASB_BOVIN	YQEPVLGPV	1000,5229	937630	1047700
sp|P02668var|CASK_VARI_BOVIN	GPFPII	642,3741	986450	954950
sp|P02666|CASB_BOVIN	YQEPVLGPVRGPFPI	1667,9035	681380	952050
sp|P02666|CASB_BOVIN	VLPVPQ	651,39556	970760	950240
P02662|CASA1_BOVIN	VAPFPEV	757,40104	862270	907710
sp|P02666|CASB_BOVIN	FVAPFPEV	904,46945	777340	882820
sp|P81265|PIGR_BOVIN	NIPPLTQTPVVVPPFLQPEV	2184,2194	224520	705780
P02662|CASA1_BOVIN	YQEPVLGPVRGPFPII	1780,9876	397690	603930
P02662|CASA1_BOVIN	SLPQNIPPLTQTPVVVPPFLQPEVM	2740,4874	19468	589960
P02754|LACB_BOVIN;sp|P02754|LACA_BOVIN	PQNIPPLTQTPVVVPPFLQPEV	2409,3308	191330	569670
sp|P02663|CASA2_BOVIN	GPVRGPFPIIV	1150,6863	377380	373970
P02662|CASA1_BOVIN	PQNIPPLTQTPVVVPPFLQPEVM	2540,3713	85294	335250
sp|P02668var|CASK_VARI_BOVIN	NIPPLTQTPVVVPPFLQPEVM	2315,2599	75195	317530
sp|P02666|CASB_BOVIN	SLPQNIPPLTQTPV	1503,8297	76272	299980
sp|P02666|CASB_BOVIN	GYGGVSLPEWV	1162,5659	177070	279990
sp|P02668var|CASK_VARI_BOVIN	GGVSLPEWV	942,48108	188470	228350
sp|P02666|CASB_BOVIN	WMHQPHQPLPPTV	1566,7766	233800	193630
sp|P02668var|CASK_VARI_BOVIN	QGPIVLNPWDQV	1364,7089	93099	182090

P02754|LACB_BOVIN;sp|P02754|LACA_BOVIN	TLTDVENLHLPLPL	1573,8716	414170	167670
sp|P02666|CASB_BOVIN	VYPFPGPIPNSLPQNIPPLTQT	2389,2682	368340	147370
P02662|CASA1_BOVIN	LYQGPIVLNPWDQV	1640,8562	79716	143470
sp|P02666|CASB_BOVIN	HNSLPQNIPPLTQT	1558,8104	175460	135610
tr|T1T0C1|VARIA1_BOVIN	SLPQNIPPLTQTPVVVPPF	2043,1405	70728	134530
P02662|CASA1_BOVIN	VYPFPGPIPNSLPQ	1524,7977	76496	134160
P02754|LACB_BOVIN;sp|P02754|LACA_BOVIN	YQEPVLGPVRGPFPIIV	1880,056	101970	130310
sp|P02668var|CASK_VARI_BOVIN	VRSPAQILQ	1010,5873	121210	116860
sp|P02668var|CASK_VARI_BOVIN	YQGPIVLNPW	1185,6182	78817	116400
sp|P02666|CASB_BOVIN	QSLVYPFPGPIPN	1427,7449	83455	114450
sp|P02666|CASB_BOVIN	LINNQFLPYPY	1380,7078	65692	112140
sp|P02666|CASB_BOVIN	VAPFPEVF	904,46945	61230	108360
sp|P02666|CASB_BOVIN	PVVVPPF	753,44251	195970	106690
P02662|CASA1_BOVIN	NPWDQV	757,3395	123920	103420
sp|P02666|CASB_BOVIN	LTDVENLHLPLPL	1472,8239	52611	97791
sp|P02663|CASA2_BOVIN	PVRGPFPIIV	1093,6648	105040	97362
P02662|CASA1_BOVIN	VYPFPGPIHNSLPQNIPPLTQT	2429,2743	463230	96885
P02662|CASA1_BOVIN	NNQFLPYPY	1154,5397	59394	95991
sp|P02663|CASA2_BOVIN	PQNIPPLTQTPVVVPPFLQPE	2310,2624	52322	95089
sp|P02668var|CASK_VARI_BOVIN	NIPPLTQTPVVVPPFLQPE	2085,151	43812	94848
sp|P02668var|CASK_VARI_BOVIN	SLPQNIPPLTQTPVVVPPFLQ	2284,2831	25808	92954
sp|P02663|CASA2_BOVIN	LHLPLPL	801,51126	95873	90687
sp|P02666|CASB_BOVIN	TEIPTINT	887,46001	109260	90642
sp|P02668var|CASK_VARI_BOVIN	PVLGPVRGPFPII	1360,8231	70734	90597
sp|P02663|CASA2_BOVIN	VLNPWDQV	969,49198	58106	89532
sp|P02666|CASB_BOVIN	FPPQSV	673,34353	69610	83979
sp|P02668var|CASK_VARI_BOVIN	PIVLNPWDQV	1179,6288	56739	79839
sp|P02668var|CASK_VARI_BOVIN	SLPQNIPPL	977,55458	44161	77967
sp|P02666|CASB_BOVIN	QFLPYPY	926,4538	66053	77807
sp|P02668var|CASK_VARI_BOVIN	WMHQPHQPLPPTVM	1697,817	81984	77705
P02662|CASA1_BOVIN	YQEPVLGPVRGPF	1457,7667	61987	74572
P02662|CASA1_BOVIN	SLTLTDVENL	1103,571	32829	71355
P02662|CASA1_BOVIN	HQGLPQEV	906,45593	87565	71090

tr|T1T0C1|VARIA1_BOVIN	YQGPIVL	788,44324	81593	65949
sp|P02668var|CASK_VARI_BOVIN	MHQPHQPLPPTVM	1511,7377	85339	65745
sp|P02666|CASB_BOVIN	LINNQFLPYP	1217,6445	39972	65701
sp|P02663|CASA2_BOVIN	LVYPFPGPIHNSLPQ	1677,8879	35122	63999
tr|T1T0C1|VARIA1_BOVIN	QEPVLGPVRGPFPI	1504,8402	30359	62003
sp|P02666|CASB_BOVIN	PVVVPPFLQPEVMGV	1606,8793	31827	58031
P02662|CASA1_BOVIN	LSFNPTQL	918,48108	46045	57216
P02662|CASA1_BOVIN	PVLGPVRGPFPIIV	1459,8915	25901	51782
P02662|CASA1_BOVIN	YVPLGTQ	776,40685	77871	50177
sp|P02668var|CASK_VARI_BOVIN	VVVPPFLQPEVM	1353,7366	30063	46434
sp|P02668var|CASK_VARI_BOVIN	VAPFPEVFGKE	1218,6285	19001	44949
sp|P02668var|CASK_VARI_BOVIN	DAYPSGAW	865,36063	51907	44787
P02662|CASA1_BOVIN	YQEPVLG	804,40177	66483	44209
sp|P02668var|CASK_VARI_BOVIN	LVYPFPGPIPNSLPQNIPPLTQT	2502,3522	15254	43590
P02754|LACB_BOVIN;sp|P02754|LACA_BOVIN	SLVYPFPGPI	1088,5906	30655	43424
sp|P02668var|CASK_VARI_BOVIN	SLPQNIPPLTQTPVVVPPFLQPE	2510,3785	61513	42430
sp|P02668var|CASK_VARI_BOVIN	MHQPHQPLPPTVMFPPQSV	2167,0707	15519	41229
sp|P02666|CASB_BOVIN	QTPVVVPPFLQPEV	1548,8552	29903	39626
sp|P02666|CASB_BOVIN	YQKFPQ	809,40719	73041	38852
P02754|LACB_BOVIN;sp|P02754|LACA_BOVIN	VVVPPFLQPEV	1222,6962	34207	38045
P02662|CASA1_BOVIN	ALPMHI	680,36797	52239	36868
P02662|CASA1_BOVIN	EPMIGV	644,32035	51642	35462
sp|P02663|CASA2_BOVIN	VVPPFLQPE	1024,5593	18933	35021
sp|P02666|CASB_BOVIN	LVYPFPGPIPNS	1299,6863	18361	32757
tr|T1T0C1|VARIA1_BOVIN	LPYPY	651,32681	43153	31576
P02662|CASA1_BOVIN	LQDKIHPF	996,53927	45266	31517
sp|P02666|CASB_BOVIN	QTPVVVPPFLQPEVM	1679,8957	23173	30322
sp|P02666|CASB_BOVIN	NIPPLTQTPVVVPPF	1617,913	16908	29777
P02662|CASA1_BOVIN	EPMIGVNQEL	1128,5485	10537	29747
sp|P02668var|CASK_VARI_BOVIN	ILDKVGIN	870,51747	31062	29065
sp|P02666|CASB_BOVIN	TKVIPYV	818,49019	21234	27948
P02662|CASA1_BOVIN	VYPFPGPIHNS	1226,6084	36069	27066
sp|P02666|CASB_BOVIN	NIPPLTQTPV	1078,6023	16196	26874

sp|P02668var|CASK_VARI_BOVIN	AMKPWI	744,39927	36152	26861
P02662|CASA1_BOVIN	SLPQNIPPLTQ	1206,6608	26985	26414
sp|P02666|CASB_BOVIN	AYFYPE	788,33811	28074	25960
sp|P02663|CASA2_BOVIN	SRYPSYG	828,37662	52355	25943
tr|T1T0C1|VARIA1_BOVIN	TDVENLHLPLPLL	1472,8239	14719	25880
sp|P02666|CASB_BOVIN	LGYLEQL	834,44872	13282	25439
sp|P02666|CASB_BOVIN	FVAPFPE	805,40104	26930	24295
P02662|CASA1_BOVIN	YYVPLG	710,36393	42747	24201
P02662|CASA1_BOVIN	SPAQIL	627,35918	43941	24119
sp|P02666|CASB_BOVIN	LYQGPIVL	901,5273	18854	24031
sp|P02668var|CASK_VARI_BOVIN	LIVTQTM	804,44153	36446	23877
tr|T1T0C1|VARIA1_BOVIN	VVPPFLQPEVM	1254,6682	86432	23606
sp|P02666|CASB_BOVIN	APFPEVFG	862,4225	17056	23116
sp|P02666|CASB_BOVIN	QFLPYP	763,39048	21497	22997
P02662|CASA1_BOVIN	GPIVLNPWDQV	1236,6503	19522	22854
P02662|CASA1_BOVIN	QSLVYPFPGPI	1216,6492	32183	22687
P02662|CASA1_BOVIN	QKEPMIGV	900,47389	27477	22649
sp|P02666|CASB_BOVIN	NQFLPYPY	1040,4967	9149,2	22441
sp|P02666|CASB_BOVIN	NIPPLTQTPVVVPPFLQ	1859,0557	7156,4	22407
sp|P02666|CASB_BOVIN	VYPFPGPIPNSLPQNIPPLT	2160,1619	15541	22374
P02754|LACB_BOVIN;sp|P02754|LACA_BOVIN	SLVYPFPGPIHN	1339,6925	9770,9	22013
sp|P02666|CASB_BOVIN	TKIPAVF	774,46398	27197	21177
sp|P02666|CASB_BOVIN	LYQGPIV	788,44324	32686	21015
sp|P02663|CASA2_BOVIN	FVAPFPEVF	1051,5379	12096	20530
P02662|CASA1_BOVIN	QEPVLGPV	837,45962	17985	20283
sp|P02668var|CASK_VARI_BOVIN	FLPYP	635,3319	27726	20188
sp|P02666|CASB_BOVIN	EPMIGVN	758,36328	17408	19925
sp|P02668var|CASK_VARI_BOVIN	ENLHLPLPL	1044,5968	16696	19331
P02662|CASA1_BOVIN	PFPGPIPN	837,43849	20309	18820
P02662|CASA1_BOVIN	NIPPLTQTPVVVPPFL	1730,9971	9533,4	17934
P02662|CASA1_BOVIN	QGLPQEV	769,39702	11649	16998
sp|Q95114|MFGM_BOVIN	MHQPHQPLPPTV	1380,6972	256690	16743
sp|P02666|CASB_BOVIN	QGLPQEVL	882,48108	13805	16737

P02662|CASA1_BOVIN	LYQGPIVLNPW	1298,7023	7761	16582
sp|P02666|CASB_BOVIN	LNVPGEIVE	968,51786	7710,9	16392
P02754|LACB_BOVIN;sp|P02754|LACA_BOVIN	YYVPLGTQ	939,47018	9448,2	16356
P02662|CASA1_BOVIN	QEPVLGPVRGPFPII	1617,9243	10688	15485
sp|P02663|CASA2_BOVIN	HQPHQPLPPTVM	1380,6972	96874	14988
P02662|CASA1_BOVIN	TPVVVPPFLQPEV	1420,7966	11271	14980
sp|Q95114|MFGM_BOVIN	DMPIQ	602,2734	41556	14880
tr|T1T0C1|VARIA1_BOVIN	LGPVRGPFPI	1051,6179	8830,5	14431
sp|P02666|CASB_BOVIN	DVENLHLPLPLL	1371,7762	7025,4	14171
P02662|CASA1_BOVIN	YQKFPQYL	1085,5546	11836	13790
sp|P02666|CASB_BOVIN	HKEMPFPK	1012,5164	7946,6	13783
sp|P02666|CASB_BOVIN	MHQPHQPLPPT	1281,6288	31645	13566
P02754|LACB_BOVIN;sp|P02754|LACA_BOVIN	FSDIPNPI	901,45453	13878	13400
sp|P02668var|CASK_VARI_BOVIN	WMHQPHQPLPPT	1467,7081	136880	13256
P02754|LACB_BOVIN;sp|P02754|LACA_BOVIN	YVPLGT	648,34828	21532	12963
sp|P02666|CASB_BOVIN	PVVVPPFLQP	1091,6379	24061	12589
sp|P02666|CASB_BOVIN	SLPQNIPPLTQTPVVVPPFL	2156,2245	24712	12157
sp|P02666|CASB_BOVIN	ALPQYL	703,39048	7322,6	12028
sp|P02666|CASB_BOVIN	YQEPVLGPVR	1156,6241	9222,2	11995
sp|P02666|CASB_BOVIN	GLPQEV	641,33844	15625	11768
P02662|CASA1_BOVIN	VLPVPQK	779,49052	23677	10557
P02754|LACB_BOVIN;sp|P02754|LACA_BOVIN	IPIQYV	731,42178	8608,5	10376
sp|P02666|CASB_BOVIN	FFSDKIA	826,4225	8657,1	10223
P00711|LALBA_BOVIN	MAIPPK	655,37272	61049	10010
sp|P02666|CASB_BOVIN	QVLSNTVPA	927,50255	15481	9759,4
sp|P02666|CASB_BOVIN	SRYPS	608,29182	18672	9732,9
sp|P02663|CASA2_BOVIN	VAPFPE	658,33263	14735	9513,7
sp|P02666|CASB_BOVIN	INNQFLP	844,4443	7945,5	9085,5
sp|P02663|CASA2_BOVIN	SRYPSY	771,35515	18118	8983,8
sp|P02666|CASB_BOVIN	HQGLPQ	678,34492	28402	8826,2
sp|P02666|CASB_BOVIN	SLPEWV	729,36974	7440	8711,8
sp|P02666|CASB_BOVIN	LIVTQTMK	932,53649	8838,9	8643,1
P02662|CASA1_BOVIN	VLVLDTDYK	1064,5754	7605,4	7987,7

sp|P02668var|CASK_VARI_BOVIN	VYPFPGPIH	1025,5335	18685	7863,2
sp|P02666|CASB_BOVIN	FFVAPFPEVFG	1255,6277	2863,4	7812
P00711|LALBA_BOVIN	QKFPQYL	922,49125	7733,4	7304,7
sp|P02666|CASB_BOVIN	ALNEINQ	800,40283	6478,4	6703,7
sp|P02666|CASB_BOVIN	HQPHQPLPPTV	1249,6568	8625,1	6056,7
P02662|CASA1_BOVIN	PFPGPIHNSLPQ	1302,6721	8643,6	6056,5
P02662|CASA1_BOVIN	NAVPIT	613,34353	25209	5592,7
sp|P02666|CASB_BOVIN	KIHPF	640,36968	9005,1	5412,8
sp|P02666|CASB_BOVIN	YQQKPVA	832,4443	9407,1	5061,2
sp|P02666|CASB_BOVIN	HNSLPQ	694,33984	7325,3	4554,6
sp|P02666|CASB_BOVIN	NMAINPS	745,34287	16139	4518,7
sp|P02666|CASB_BOVIN	KAVPYPQ	801,43849	6812,7	4294,5
sp|P02666|CASB_BOVIN	YYQQKPVA	995,50763	9820,2	3770,2
sp|P02666|CASB_BOVIN	VPLGTQ	613,34353	6179,7	3373,3

Peptides only in CON

P02662|CASA1_BOVIN	VAPFPEVFG	961,49092	1280900	0
P02662|CASA1_BOVIN	INNQFLPYPY	1267,6237	1246100	0
sp|P02666|CASB_BOVIN	VYPFPGPIHNSLPQ	1564,8038	1159100	0
sp|P02666|CASB_BOVIN	AVPYPQ	673,34353	566630	0
P02754|LACB_BOVIN;sp|P02754|LACA_BOVIN	VENLHLPLPL	1143,6652	420210	0
sp|P02666|CASB_BOVIN	DMPI	474,21482	152650	0
P02662|CASA1_BOVIN	SPAQILQ	755,41775	123880	0
sp|P02663|CASA2_BOVIN	TLTDVENL	903,45493	112590	0
sp|P02668var|CASK_VARI_BOVIN	FPPQSVL	786,42759	77242	0
P02662|CASA1_BOVIN	FSDKIA	679,35409	61631	0
sp|P02668var|CASK_VARI_BOVIN	HQGLPQEVL	1019,54	57427	0
sp|P02668var|CASK_VARI_BOVIN	LYQEPVLGPV	1113,607	56275	0
sp|P02666|CASB_BOVIN	INNQFLPY	1007,5076	54188	0
sp|P02666|CASB_BOVIN	YKVPQ	633,34861	49572	0
sp|P02666|CASB_BOVIN	YAKPAA	619,33296	39064	0
tr|T1T0C1|VARIA1_BOVIN	VYPFPGPIHNSLPQNIPPLT	2200,1681	30706	0
sp|P02668var|CASK_VARI_BOVIN	YKVPQLEIVPN	1298,7234	28658	0

sp|P02663|CASA2_BOVIN	PQNIPPLTQTPVVVPPFLQ	2084,167	28181	0
sp|P02666|CASB_BOVIN	SRYPSYGLN	1055,5036	24275	0
P02754|LACB_BOVIN;sp|P02754|LACA_BOVIN	YQGPIVLNPWDQ	1428,7038	22489	0
sp|P02663|CASA2_BOVIN	SNTVPA	587,29149	22412	0
sp|P02666|CASB_BOVIN	LVYPFPGPIHNSLPQNIPPLTQT	2542,3584	20596	0
P02662|CASA1_BOVIN	PQNIPPLTQTPVVVPPF	1843,0244	19831	0
sp|P02666|CASB_BOVIN	TDVENL	689,32318	17929	0
sp|P02666|CASB_BOVIN	VRSPAQI	769,44464	17117	0
sp|P02666|CASB_BOVIN	NNQFLPYP	991,47633	14986	0
sp|P02666|CASB_BOVIN	YQEPV	634,29624	13965	0
sp|P02668var|CASK_VARI_BOVIN	LNVPGE	627,32279	13256	0
sp|P02663|CASA2_BOVIN	QDKIHPF	883,4552	12103	0
P02662|CASA1_BOVIN	NIFETPF	866,41742	12054	0
sp|P02666|CASB_BOVIN	YPSYGLN	812,37047	11940	0
tr|T1T0C1|VARIA1_BOVIN	AMKPW	631,3152	11236	0
sp|P02663|CASA2_BOVIN	YYQQKPV	924,47052	11217	0
sp|P02663|CASA2_BOVIN	LYQEPVLG	917,48583	11064	0
sp|P02666|CASB_BOVIN	VYPFPGPIHN	1139,5764	11040	0
sp|P02666|CASB_BOVIN	KTTMPL	689,3782	10594	0
sp|P02666|CASB_BOVIN	FFVAPFPEV	1051,5379	10591	0
sp|P02668var|CASK_VARI_BOVIN	SDIPNPIG	811,40758	9494,7	0
tr|T1T0C1|VARIA1_BOVIN	YVPLGTQY	939,47018	9448,2	0
sp|P02663|CASA2_BOVIN	FFAKESV	826,4225	8657,1	0
sp|P02668var|CASK_VARI_BOVIN	SLLDAQ	645,33336	7712,8	0
sp|P02663|CASA2_BOVIN	RNAVPITPT	967,54508	7291,1	0
sp|P02666|CASB_BOVIN	AVPITPT	697,40104	6015,5	0
sp|P02666|CASB_BOVIN	SFNPTQL	805,39702	5300,7	0
tr|T1T0C1|VARIA1_BOVIN	LIVTQT	673,40104	5214,1	0
sp|P02666|CASB_BOVIN	VLSNTVPA	799,44397	4170,7	0
P00711|LALBA_BOVIN	YTDAPS	652,27042	4035,5	0
P02662|CASA1_BOVIN	DAYPSGA	679,28132	3271	0
sp|P02666|CASB_BOVIN	IVTQTM	691,35746	3234,6	0
sp|P02666|CASB_BOVIN	DAYPSG	608,24421	2758,9	0

sp|P02666|CASB_BOVIN	QFYQ	584,25946	2732,3	0
sp|P02666|CASB_BOVIN	RQFYQ	740,36057	1998,8	0

Peptides only in OOP

sp|P02666|CASB_BOVIN	FVAPFPEVFG	1108,5593	0	1180800
tr|T1T0C1|VARIA1_BOVIN	YPVEPF	750,35884	0	1072300
sp|P02666|CASB_BOVIN	DVENLHLPLPL	1258,6921	0	984410
sp|P02668var|CASK_VARI_BOVIN	PVLGPVRGPFPI	1247,739	0	426830
tr|T1T0C1|VARIA1_BOVIN	EMPFPK	747,36255	0	271720
sp|P02668var|CASK_VARI_BOVIN	SDIPNPI	754,38612	0	261930
P02662|CASA1_BOVIN	PVVVPPFLQ	994,58515	0	233640
sp|P02666|CASB_BOVIN	LVYPFPGPI	1001,5586	0	216220
P02662|CASA1_BOVIN	PVRGPFPII	994,59639	0	126120
sp|P02666|CASB_BOVIN	TKVIPY	719,42178	0	100250
sp|P02663|CASA2_BOVIN	LVYPFPGPIHN	1252,6604	0	79505
sp|P02666|CASB_BOVIN	TQTPVVVPPFLQPEV	1649,9029	0	56649
P00711|LALBA_BOVIN	NIPPLTQTPVVVPPFLQPEVMG	2372,2814	0	54176
sp|P02663|CASA2_BOVIN	PVVVPPFLQPEVMGVS	1693,9113	0	48599
sp|P02666|CASB_BOVIN	ILDKVGI	756,47454	0	46353
sp|P02666|CASB_BOVIN	EPMIGVNQELA	1199,5856	0	45933
sp|P02663|CASA2_BOVIN	PQNIPPLTQT	1107,5924	0	39238
P02662|CASA1_BOVIN	PQNIPPLTQTPVVVPPFLQPEVMG	2597,3927	0	36750
sp|P02666|CASB_BOVIN	SDIPNPIGSENSEK	1485,6947	0	35939
sp|P02663|CASA2_BOVIN	TQTPVVVPPFLQPEVM	1780,9433	0	33271
sp|P02668var|CASK_VARI_BOVIN	VEELKPTPEGDLEIL	1680,8822	0	27600
sp|P02668var|CASK_VARI_BOVIN	TKIPAV	627,39556	0	25125
P00711|LALBA_BOVIN	EGIHAQQKEPMIGV	1535,7766	0	21994
P02662|CASA1_BOVIN	TPVVVPPFLQPEVM	1551,8371	0	21765
sp|P02668var|CASK_VARI_BOVIN	YQGPIVLNPWDQVK	1655,8671	0	21083
sp|P02666|CASB_BOVIN	GLDIQKVAGTWY	1349,698	0	20414
P02662|CASA1_BOVIN	SLPQNIPPLTQTP	1404,7613	0	19948
sp|P02663|CASA2_BOVIN	LYQEPVLGPVRGPFPI	1780,9876	0	18529
sp|P02666|CASB_BOVIN	TQTPVVVPPFLQPE	1550,8344	0	16742

sp|P02666|CASB_BOVIN	HNSLPQNIPPLTQTPV	1754,9315	0	15766
P02662|CASA1_BOVIN	VAPFPEVFGK	1089,5859	0	15719
P02662|CASA1_BOVIN	LVYPFPGPIPNSLPQ	1637,8817	0	15095
sp|P02666|CASB_BOVIN	LTDVENL	802,40725	0	14847
P02754|LACB_BOVIN;sp|P02754|LACA_BOVIN	NQDKTEIPTINT	1372,6834	0	14319
tr|T1T0C1|VARIA1_BOVIN	RELEEL	787,40758	0	13387
sp|P02666|CASB_BOVIN	FLDDDLTDDI	1180,5136	0	11864
sp|P02668var|CASK_VARI_BOVIN	LTLTDVENL	1016,539	0	11800
sp|P02666|CASB_BOVIN	VQVT	445,25365	0	11339
sp|P02668var|CASK_VARI_BOVIN	IFIGNVNNSGL	1146,6033	0	10745
sp|P02666|CASB_BOVIN	YLGYLEQ	884,42798	0	9084,9
sp|P02666|CASB_BOVIN	PMIGVNQELA	1070,543	0	8888,1
sp|P02663|CASA2_BOVIN	VYPFPGPI	888,47454	0	8787,3
sp|P02666|CASB_BOVIN	ESPPEINT	885,40798	0	7822,4
P02662|CASA1_BOVIN	KNQDKTEIPTINT	1500,7784	0	7743
sp|P02666|CASB_BOVIN	IGVNQELA	842,44978	0	7500,9
sp|P02668var|CASK_VARI_BOVIN	QKEPMIGVNQELA	1455,7392	0	6409,9
sp|P02666|CASB_BOVIN	QKEPMIGVNQEL	1384,7021	0	6058,9
sp|P02666|CASB_BOVIN	IASGEPTSTPTIE	1301,6351	0	5983,9
sp|P02663|CASA2_BOVIN	LEIVPN	683,38539	0	5199
sp|P02666|CASB_BOVIN	QKEPMIGVN	1014,5168	0	5054,1
sp|P02666|CASB_BOVIN	SLTLTDVE	876,44403	0	4667,1
sp|P02663|CASA2_BOVIN	FYPE	554,23766	0	4545,3
sp|P02666|CASB_BOVIN	ITVDDKHYQ	1117,5404	0	4163,2
sp|P02666|CASB_BOVIN	YQQKPV	761,40719	0	3808,3
P00711|LALBA_BOVIN	HIQKEDVPSE	1180,5724	0	3035,2
sp|P02666|CASB_BOVIN	EGIHAQQ	781,37187	0	2498,1
sp|P02666|CASB_BOVIN	VPLGT	485,28495	0	2012,3
sp|P02666|CASB_BOVIN	STVAT	477,24348	0	1941,2
sp|P02666|CASB_BOVIN	SPAQI	514,27511	0	1662,1

°Uniprot ID
